# Home-Based Transcranial Direct Current Stimulation for the Treatment of Symptoms of Depression and Anxiety in Temporal Lobe Epilepsy: A Randomized, Double-Blind, Sham-Controlled Clinical Trial

**DOI:** 10.3389/fnint.2021.753995

**Published:** 2021-12-08

**Authors:** Suelen Mandelli Mota, Luiza Amaral de Castro, Patrícia Gabriela Riedel, Carolina Machado Torres, José Augusto Bragatti, Rosane Brondani, Thais Leite Secchi, Paulo Roberto Stefani Sanches, Wolnei Caumo, Marino Muxfeldt Bianchin

**Affiliations:** ^1^Programa de Pós-Graduação em Ciências Médicas, Universidade Federal do Rio Grande do Sul, Porto Alegre, Brazil; ^2^Universidade Federal do Rio Grande do Sul, Porto Alegre, Brazil; ^3^Centro para Tratamento de Epilepsia Refratária (CETER), Basic Research and Advanced Investigations in Neuroscience (BRAIN), Serviço de Neurologia do Hospital de Clínicas de Porto Alegre, Porto Alegre, Brazil; ^4^Laboratório de Engenharia Biomédica, Universidade Federal do Rio Grande do Sul, Porto Alegre, Brazil; ^5^Laboratório de Dor & Neuromodulação, Hospital de Clínicas de Porto Alegre, Porto Alegre, Brazil

**Keywords:** tDCS – transcranial direct current stimulation, epilepsy, depression, anxiety, neuromodulation, non-pharmacological interventions

## Abstract

We conducted a double-blind randomized clinical trial in order to examine the effects and the safety of home-based transcranial direct current stimulation (tDCS) on depressive and anxious symptoms of patients with temporal lobe epilepsy (TLE). We evaluated 26 adults with TLE and depressive symptoms randomized into two different groups: active tDCS (tDCSa) and Sham (tDCSs). The patients were first submitted to 20 sessions of tDCS for 20 min daily, 5 days a week for 4 weeks and then received a maintenance tDCS application in the research laboratory once a week for 3 weeks. The intensity of the current was 2 mA, applied bilaterally over the dorsolateral prefrontal cortex, with the anode positioned on the left side and the cathode on the right side. Participants were evaluated on days 1, 15, 30, and 60 of the study using the Beck Depression Inventory II (BDI). A follow-up evaluation was performed 1 year after the end of treatment. They were also evaluated for quality of life and for anxious symptoms as secondary outcomes. The groups did not differ in clinical, socioeconomic or psychometric characteristics at the initial assessment. There was no statistically significant difference between groups regarding reported adverse effects, seizure frequency or dropouts. On average, between the 1st and 60th day, the BDI score decreased by 43.93% in the active group and by 44.67% in the Sham group (ΔBDIfinal – initial = −12.54 vs. −12.20, *p* = 0.68). The similar improvement in depressive symptoms observed in both groups was attributed to placebo effect and interaction between participants and research group and not to tDCS intervention *per se*. In our study, tDCS was safe and well tolerated, but it was not effective in reducing depressive or anxiety symptoms in patients with temporal lobe epilepsy.

**Clinical Trial Registration:** [ClinicalTrials.gov], identifier [NCT03871842].

## Introduction

Temporal lobe epilepsy (TLE) is one of the most frequent forms of focal epilepsy and is associated with high rates of neuropsychiatric disorders (Bragatti et al., 2011). Depressive and anxiety disorders are the main comorbid neuropsychiatric disorders in patients with epilepsy and TLE (Kwon and Park, 2014). Depressive and anxiety disorders can impair the quality of life of patients with epilepsy (PWE) and can increase adverse events related to the use of antiepileptic drugs (AEDs) (Kanner et al., 2012; Kwon and Park, 2014). It has been shown that not only the control of crises, but also the treatment of psychiatric comorbidities is important for the quality of life of epilepsy patients (Boylan et al., 2004; Mahrer-Imhof et al., 2013; Ertem et al., 2017).

Transcranial Direct Current Stimulation (tDCS) has been studied as a complementary and safe therapeutic option for the treatment of psychiatric disorders in PWE (Liu et al., 2016). There is evidence that depressive and anxiety disorders (with the exception of generalized anxiety disorder) are associated with cortical hypoactivity in the left cerebral hemisphere and increased cortical excitability in the right cerebral hemisphere (Nitschke and Heller, 2005; Grimm et al., 2008). Thus, although the results of treatment with tDCS in psychiatric disorders are still contrasting (Nitsche et al., 2009; Gomes et al., 2019; Stein et al., 2020), the positioning of the anode (excitatory) electrode on the left dorsolateral prefrontal cortex (DLPFC) and the cathode (inhibitory) electrode on the right DLPFC can be promising in improving depressive symptoms and anxiety (Boggio et al., 2008; Loo and Martin, 2012; Brunoni et al., 2017; Stein et al., 2020).

Some studies on tDCS in the treatment of depressive disorder and anxiety have used the anodal stimulation protocol over the left DLPFC, with the cathode electrode positioned over the right DLPFC, with 2mA intensity, for 20 to 30 min a day for 5 to 30 days (Boggio et al., 2008; Loo et al., 2012; Stein et al., 2020). However, the only previous study that used anodic tDCS on the left DLPFC for the treatment of depressive symptoms of patients with TLE (5 sessions, 2mA) evaluated minimal depressive symptoms, obtaining an average reduction of only 1.68 points in the Beck Depression Inventory-II (BDI) in the group that received active tDCS, and an average increase of 1.27 points in the Sham group (Liu et al., 2016). We propose to study the effect of tDCS on depressive symptoms considered at least mild (minimum BDI score of 14 points).

Considering that a longer treatment time can produce better results in depressive and anxiety symptoms (Padberg et al., 2017; de Lima et al., 2019) and that, according to clinical and experimental evidence, conventional models of tDCS are not associated with the generation of epilepsy crises (Bikson et al., 2016; Liu et al., 2016), we propose to expand the treatment of depressive symptoms of patients with TLE using 23 sessions of bimodal home-based tDCS (anode positioned over the left DLPFC and cathode over the right DLPFC). In order to make the prolonged use of the device by the participants viable, we propose the innovative use of the self-administered home-based and self-administered tDCS (Carvalho et al., 2018).

This study aim to contribute to a better understanding of the potential of tDCS in patients with epilepsy and eventually assist in the development of new treatment protocols for depressive and anxiety disorders in patients with TLE.

## Materials and Methods

### Ethics

The present study was conducted in accordance with the World Medical Association’s code of ethics, the Declaration of Helsinki and the rules established by the Research Ethics Committee of the Hospital de Clínicas de Porto Alegre, under the Institutional Review Board CAAE 83801517100005327. All participants signed an informed consent form, which describes that participants will be allocated to a sham or active stimulation group, it describes also the mechanism of action of the tDCS as well as its possible benefits, possible side effects and contraindications.

### Subjects

The clinical trial was carried out at the Hospital de Clínicas de Porto Alegre epilepsy clinic between January 2019 and March 2020, involving a group of 26 adults (>18 years-old) diagnosed with TLE according to the International League Againt Epilepsy (ILAE) classification for epileptic seizures and syndromes. All patients had depressive symptoms as evaluated by the Structured Clinical Interview for DSM-4 (≥ 4 points in the BDI) and were able to adequately answer the questionnaires and to be able to undergo the treatment. Exclusion criteria were: (1) failure to give informed consent; (2) change in the antiepileptic drug regimen (AED) or in the antidepressant medication regimen 30 days before or during the study; (3) history of status epilepticus in the previous year; (4) being submitted to vagus nerve stimulation, deep brain stimulation or any other type of neurostimulation less than 1 year before this study; (5) active suicidal ideation; (6) contraindication for tDCS, including presence of any metal on the head or any implanted electrical medical device such as pacemakers and cardiac defibrillators, and (7) pregnancy. To ensure adequate adherence, correct use of the device and adequate responses to the questionnaires, we only included patients with normal I.Q. and that we considered that would understand the study.

### Calculation of Sample Size

One of the few studies on tDCS for the treatment of depressive symptoms in PWE was conducted on patients with mild or minimal depressive symptoms, obtaining a very small improvement of these symptoms when applying the tDCS for 5 days (−1.68 vs. + 1.27 points in the BDI in the active tDCS vs. Sham group, respectively) (Liu et al., 2016). Hence, we based our sample size calculation on a study with a methodology similar to ours, even if applied to patients without epilepsy, but with moderate or severe depressive symptoms, who received intervention of at least 3 weeks with anodal tDCS (Loo et al., 2012).

The sample size was calculated using the WinPepi program to detect differences in mean BDI score over time, between the tDCS Active and Sham groups. Considering 80% power, a 5% level of significance, an expected difference of 9 points for the active tDCS group, and a standard deviation of 7.1 points for the active group and of 7.9 points for the Sham group (Loo et al., 2012), the sample size obtained was 22 subjects, divided into two symmetrical groups. After adding approximately 15% for possible losses, the sample size included at least 26 subjects.

### Randomization and Blinding

Considering that this was a parallel study, the participants were stratified according to the laterality of their epilepsy crises (left, right or bilateral) and were then randomized into blocks of 4 using appropriate software and respecting the 1:1 allocation ratio. The only researcher who had access to the randomization list was the biomedical engineer responsible for configuring the tDCS equipment to active or Sham. All researchers involved in conducting the interviews, contacting the participants and analyzing the data, as well as all included participants were blinded to the allocation. To ensure the participant’s blinding, Sham pacing was programmed to provide 30 s of progressive pacing (15 s 0–2 mA and 15 s 2–0 mA) at the beginning, middle and end of the application to promote the tactile effects of electric current. At the end of the study, we asked the participants what type of stimulation they believed to have received and what was their confidence in the response using a Likert scale from 1 (without confidence) to 5 (total confidence).

### Intervention

We developed a 2-month treatment protocol. The number of tDCS sessions was chosen based on previous articles (Boggio et al., 2008; Loo et al., 2012; Padberg et al., 2017; de Lima et al., 2019; Stein et al., 2020) and was adapted to the logistics available at the research center of our Institution. In this study, we used the following protocol for tDCS: anodic and cathodic electrodes respectively positioned over the left and right DLPFC, 2mA electric current intensity, stimulation lasting 20 min per day and applied daily for 4 consecutive weeks, with breaks on weekends, followed by 3 sessions held once a week. These sessions were done at our center and not home-based. During the study design, we questioned the possibility that the effect of daily treatment with tDCS for 1 month persists with weekly stimulations for another 3 weeks. The number of 3 weekly sessions applied at research center and not at home were defined due to our logistical possibilities of treatment time, available space and exam rooms, number of participants to be recruited and number of tDCS available.

The current was supplied with 35 cm^2^ electrodes coated with a vegetable sponge moistened with saline solution before the start of stimulation by 2 silicone cannulas attached to the electrode. A medical engineer prepared the device for a fixed number of stimulations, with a minimum 16-h safety interval between 2 consecutive sessions.

We chose the home-based tDCS for home use, whose characteristics are specified in an article by Carvalho et al. (2018). This tDCS device is self-administered. The participant is trained to use the device in the first evaluation day. Then, the patients were invited to demonstrate by themselves the use of the device in the first application, to avoid misunderstandings. They received a video to watch at home and a step-to-step list. The electrodes are previously positioned and fixed in a cap in order to maintain the correct use of the electrodes by the participant. It guarantees the correct daily current delivery, even when the device is self-administered by the own participant. Details of the device and the step-by-step process for self-administration of the tDCS device at home can be viewed by visiting the link^1^.

### Procedures

**FIGURE 1:**
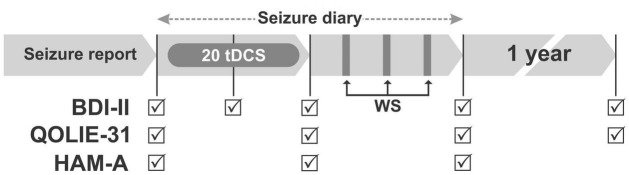
Each arrow corresponds to a 30-day period, except the last (1-year follow up). Patients self-administered 20 sessions of tDCS at home during 20 min daily (20 tDCS), 5 days a week for 4 weeks. Next, three maintenance consecutive weekly sessions (WS) of tDCS’s were applied in the research laboratory. To assess the frequency of seizures, patients filled in seizure reports during a month prior to the start of treatment and kept a seizure diary from day one to day 60 of the study. Participants were evaluated on days 1, 15, 30, 60, and after 1-year follow up of the study using the Beck Depression Inventory II (BDI II). The Inventory for Quality of Life in Epilepsy (QOLIE-31) was evaluated on days 1, 30, 60 and after 1-year follow up. The Hamilton Anxiety Scale (HAM-A) were applied only on days 1, 30 and 60.

Patients were evaluated on days 1, 15, 30, and 60 of the study. A follow-up evaluation was also performed approximately 1 year after the end of treatment. To assess the frequency of seizures, the participants reported the number of seizures during the last month before the beginning of treatment and kept a seizure diary from the first to the 60th day of the study. In the second assessment (day 30), the participants returned the equipment they had received on the first day of the study (Figure 1). All professionals involved in the interviews were trained to communicate equally with all participants. Remote supervision was available to the participants via a social network (*Whatsapp*), video and telephone calls during treatment. However, the supervision was not standardized, did not have a pre-established number of remote meeting. It was available if requested by the participant. The information stored on the device, such as number of sessions performed, impedance and duration of the sessions, was recorded by an engineer not involved in the treatment of the participants. The engineer kept these data in his care until the end of the study. Details about the representation of the study procedures over time are shown in Figure 1.

### Instruments and Assessments

The main outcome of this study was the score of depressive symptoms obtained with the Beck Depression Inventory II (BDI), one of the best self-report measures of depression, widely used in clinical research and validated for Brazil (Beck et al., 1993; Gomes-Oliveira et al., 2012). The BDI can be used to screen for depressive symptoms in PWE, with approximately 90% sensitivity and specificity to predict the diagnosis of depression (de Oliveira et al., 2014). The BDI consists of 21 items that assess the severity of depressive symptoms on a Likert scale from 0 to 3 and can be considered an economical tool for measuring the severity of depression, which is widely applicable both to research and to clinical settings all over the world (Gomes-Oliveira et al., 2012).

To assess the impact of treatment on patients’ quality of life, we used the Inventory for Quality of Life in Epilepsy (QOLIE-31) (Azevedo et al., 2009). The 31 items of this inventory are divided into seven domains: concern, apprehension, global assessment of quality of life, emotional well-being, feeling of energy or fatigue, cognitive functions, effects of medication, and social relationships. It is a self-administered questionnaire. Results are converted to a scale of 0 to 100 to determine quality of life; the lower the score, the lower the quality of life. Values below 40 in the QOLIE-31 scale reflect a poor quality of life, values between 41 and 60 reflect good self-perceived quality of life and values above 61 reflect an excellent quality of life.

Anxious symptoms were assessed using the Hamilton Anxiety Scale (HAM-A) (Hamilton, 1959). HAM-A was one of the first assessment scales developed to measure the severity of symptoms of anxiety, and is still widely used today both in clinical and research settings. The scale consists of 14 items, measures both the psychological symptoms of anxiety (mental agitation and psychological distress) and somatic anxiety (physical complaints related to anxiety) and has acceptable levels of reliability reported by evaluators. Each item is scored on a scale from 0 (absent) to 4 (severe), with a total score range of 0–56, where <17 indicates mild severity, 18–24 mild to moderate severity and 25–30 moderate to strong symptoms.

### Statistical Analysis

All analyses were performed using the IBM Statistical Analysis Software Package (SPSS), Version 20, with bilateral significance tests, at the 5% level of significance. The analyses were performed with the intention of treating. Only one participant had a baseline BDI score higher than ± 2.5 standard deviations (SD) of the group average. This outlier was not excluded. It didn’t affect the calculation of the results.

We compared the demographic, clinical and neuropsychological characteristics of the groups at the beginning of the study using the t test for continuous variables and the Chi-square test or Fisher’s exact test for categorical variables. Continuous variables were tested for normal distribution using the Shapiro-Wilk normality test. For asymmetric distributions, the groups were compared by the Wilcoxon-Mann-Whitney test. To analyze the psychometric results (BDI and QOLIE-31), we generated a model of Generalized Estimating Equations (GEE) with a dependent variable (BDI-II, QOLIE-31 or HAM-A score), and within-subject variable (time), a variable between subjects (active × Sham tDCS) and control for covariates (age, sex, education, AED, antidepressant drugs (DAD), occupation and income). Bonferroni correction was used for *post hoc* analysis.

In order to control the covariates, correlation analysis and multivariate analysis of covariance were performed between the following variables: deltaBDI (final-initial), deltaQOLIE-31 (final-initial) and deltaHAM-A (final-initial) vs. type of stimulation, age, sex, education, AED, DAD, occupation and income.

## Results

Of the 120 patients with TLE evaluated for participation in this study at the Hospital de Clínicas de Porto Alegre epilepsy clinic between February 2019 and March 2020, 35 met the inclusion and exclusion criteria. Of these, 26 agreed to participate in the study, being randomized into two symmetrical groups. In the blinding validation questionnaire, two subjects from the Sham group and one from the active group believed they had received a placebo treatment. All other participants believed they have received active intervention. In the active group, one participant dropped out of the trial due to burning discomfort and pain in the scalp produced by the equipment. In the Sham group, one participant left the trial due to loss of interest, and another participant needed to travel. There was no statistically significant difference between groups regarding dropouts from the study. No participant was excluded from the final data analysis. There was no statistically significant difference between groups regarding clinical, sociodemographic, psychometric characteristics and drug uses in the first assessment (pre-treatment) (Tables 1–3).

**TABLE 1:** Clinical and demographic characteristics of the sample.

Demographic characteristics	Active tDCS (*n* = 13)	Sham tDCS (*n* = 13)	*p* value
Age in years (mean, SD)	53.38 (±14.45)	55.76 (±7.68)	0.60
Sex female (n, %)	12 (92.30%)	10 (76.92%)	0.59
Schooling-years of study (median, IQR)	6.0 (5.0–9.0)	6.0 (5.0–10.0)	0.84
Income in number of minimum wages (median, IQR)	1.55 (1.0–3.0)	2.0 (1.0–4.0)	0.48
Occupational situation (n, %)			0.59
Unemployed	8 (61.53%)	6 (46.15%)	
Retired	5 (38.46%)	5 (38.46%)	
Sickness benefit	0 (0%)	2 (15.38%)	
From Porto Alegre	7 (53.84%)	8 (61.53%)	1.00
Received help from caregiver during treatment	11 (84.61%)	76.92%)	1.00
Laterality of the crisis (n, %)			1.00
Left	8 (61.53%)	9 (69.23%)	
Right	1 (7.69%)	0 (0%)	
Bilateral	4 (30.76%)	4 (30.73%)	
Age of onset of epilepsy (mean, SD)	22.83 (±13.06)	22.23 (±13.68)	0.91
Dropouts (n, %)	2 (15.38%)	1 (7.69%)	1.00
Use of psychiatric medications (n, %)	7 (53.84%)	7 (53.84%)	1.00
N of epilepsy seizures in the last month (median, minimum-maximum)	0 (0–1)	0 (0–2)	0.68
Controlled epilepsy (n, %)	3 (23.1%)	8 (61.5%)	0.11
Previous psychiatric diagnosis (n, %)			0.57
Mood disorder	7 (53.84%)	6 (46.15%)	1.00
Anxiety disorder	0 (0.0%)	1 (7.69%)	1.00
Psychotic disorder	0 (0.0%)	2 (15.38%)	0.48
Mood + Anxiety	3 (23.07%)	2 (15.38%)	1.00
Without disorder	1 (7.69%)	1 (7.69%)	1.00
Not described	2 (15.38%)	0 (0.0%)	1.00

**TABLE 2:** Initial psychological tests and quality of life evaluations.

Tests	Active tDCS (*n* = 13)	Sham tDCS (*n* = 13)	*p* value
BDI-II (mean, DP)	28.53 (±5.51)	27.30 (±7.29)	0.63
QOLIE-31 (mean, DP)	47.70 (±13.09)	50.54 (±13.55)	0.59
HAM-A (mean, DP)	23,23 (±8,36)	22,92 (±4,28)	0.90

**TABLE 3:** Antiepileptic and psychiatric medications in use.

Medications in use	Active tDCS (*n* = 13)	Sham tDCS (*n* = 13)	*p* value
**Antiepileptic medications**			
Valproic acid (n, %)	2 (15.4%)	5 (38.5%)	0.37
Carbamazepine (n, %)	7 (53.8%)	9 (69.2%)	0.68
Clobazam (n, %)	1 (7.7%)	1 (7.7%)	1.00
Phenytoin (n, %)	3 (23.1%)	2 (15.4%)	1.00
Phenobarbital (n, %)	3 (23.1%)	4 (30.8%)	1.00
Lamotrigine (n, %)	0 (0%)	1 (7.7%)	1.00
Oxcarbazepine (n, %)	2 (15.4%)	0 (0%)	0.48
**Psychiatric medications**			
Amitriptyline (n, %)	2 (15.4%)	1 (7.7%)	1.00
Benzodiazepine (n, %)	1 (7.7%)	1 (7.7%)	1.00
Chlorpromazine (n, %)	0 (0%)	2 (15.4%)	0.48
Fluoxetine (n, %)	5 (38.5%)	4 (30.8%)	1.00
Imipramine (n, %)	0 (0%)	2 (15.4%)	0.48
Lithium (n, %)	0 (0%)	1 (7.7%)	1.00
Risperidone (n, %)	0 (0%)	1 (7.7%)	1.00
Sertraline (n, %)	1 (7.7%)	1 (7.7%)	1.00

### Safety and Adverse Effects

The tDCS did not increase the frequency of seizures during the month of home treatment in relation to the frequency of seizures during the month prior to treatment (*p* = 0.3) (Table 4). Regarding the perception of adverse effects of the use of tDCS, 63% of the subjects who used active tDCS, and 25% of those who received tDCS Sham, reported some moderate or severe adverse effect of the use of tDCS (Table 5) such as tingling, itching or scalp redness, headache, neck pain, drowsiness, or change in concentration or mood (*X*^2^ = 1.69; *p* = 0.193) (Table 6). In the active group, one participant dropped out owing to burning discomfort and pain in the scalp produced by the equipment. In the Sham group, one participant dropped out due to loss of interest, and another participant needed to travel. Although there was no statistically significant difference between groups in terms of the reported adverse effects, seizure frequency or dropouts, the participants of both groups held only a mean of 13 of 20 sessions of tDCS at home (Table 7). Data such as average resistance and time of use of the device were recorded by the devices themselves. Sessions considered effective were those performed for more than 10 min on each day of use of the device, with an adequate record of resistance and impedance. Many evaluations were missed due to the unavailability of the participants to return to the research center. The number of participants who returned for each of the four assessments (t1, t2, t3, t4) in the active and Sham groups was, respectively: t1 (13,13); t2 (9, 11); t3 (11, 12); t4 (10, 9).

**TABLE 4:** Frequency of seizures expressed as an average number of seizures in the 30 days prior to the start of treatment, in the 30 days after the start of treatment and during the 30 days after the end of home treatment.

Crisis frequency	Active tDCS (*n* = 13)	Sham tDCS (*n* = 13)	*p* value
30 days before starting treatment (mean, SD)	0.25 (±0.45)	0.18 (±0.40)	0.55
During the 30 days of home treatment (mean, SD)	0.17 (±0.38)	0.0 (±0.0)	
Between 30 and 60 days of follow-up (mean, SD)	0.50 (±1.73)	0.0 (±0.0)	

*The numbers were compared using the generalized estimating equations.*

**TABLE 5:** Adverse effects (AE) to tDCS reported by active or sham tDCS groups.

Adverse effects	Active tDCS (*n* = 11)	Sham tDCS (*n* = 12)	*p* value
None or mild (n, %)	4 (36.6%)	9 (75%)	0.10
Moderate or severe (n, %)	7 (63.6%)	3 (25%)	

**TABLE 6:** Adverse effects of using tDCS reported by active tDCS or Sham groups.

Adverse effects (n, %)	Active tDCS (*n* = 11)			Sham tDCS (*n* = 12)			*p* value
	Mild	Moderate	Severe	Mild	Moderate	Severe	
Headache	2 (18.2%)	1 (9.31%)	(9.1%)	1(8.3%)	2 (16.7%)	0 (0%)	0.59
Neck pain	0 (0%)	2 (18.2%)	0 (0%)	0 (0%)	0 (0%)	0 (0%)	0.21
Scalp pain	0 (0%)	1 (9.1%)	0 (0%)	0 (0%)	1 (8.3%)	0 (0%)	1.00
Tingling	3 (27.3%)	1 (9.1%)	0 (0%)	1 (8.3%)	1 (8.3%)	0 (0%)	0.47
Itching	1 (9.1%)	2 (18.2%)	1 (9.1%)	2 (16.7%)	1 (8.3%)	0 (0%)	0.59
Burning	3 (27.3%)	3 (27.3%)	1 (9.1%)	4 (33.3%)	1 (8.3%)	0 (0%)	0.40
Redness	1 (9.1%)	0 (0%)	0 (0%)	0 (0%)	1 (8.3%)	0 (0%)	0.36
Somnolence	2 (18.2%)	0 (0%)	0 (0%)	1 (8.3%)	0 (0%)	1 (8.3%)	0.51
Difficulty concentrating	1 (9.1%)	0 (0%)	0 (0%)	1 (8.3%)	1 (8.3%)	0 (0%)	0.61
Mood swings	0 (0%)	1 (9.1%)	0 (0%)	0 (0%)	0 (0%)	0 (0%)	0.47
Other	0 (0%)	0 (%)	0 (0%)	0 (0%)	0 (0%)	0 (0%)	1.00

**TABLE 7:** Adherence to the use of home-based tDCS assessed by recording the number of sessions performed at home (maximum number = 20 sessions).

Adherence to the use of home-based tDCS	Active tDCS (n = 13)	Sham tDCS (n = 13)	*p* value
Number of home sessions (mean, SD)	15.38 (±4.87)	15.46 (±5.69)	0.97
No. of sessions actually held at home (mean, SD)	13.66 (±5.14)	13.07 (±7.35)	0.81

*Sessions whose electrical current maintained its stimulation for less than 10 min were disregarded, being recomputed on the second line.*

### Depressive Symptoms

**FIGURE 2:**
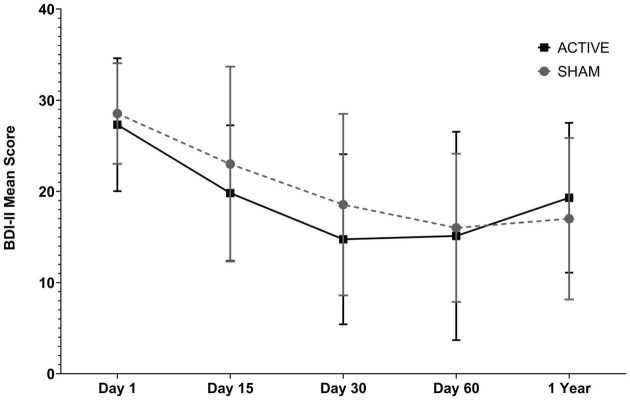
Assessment of depressive symptoms in the Sham and Active groups (using the Beck II Depression Inventory - BDI-II) during five stages (i) pre-treatment; (ii) after 10 sessions of tDCS; (iii) after 20 daily sessions, and (iv) after 1 month of follow-up performing tDCS once a week for 3 weeks; (v) 1 year after treatment. Data arereported as mean +SD score on the BDI-II scale. Data analysis was performed using the Generalized Estimating Equations model (GEE). There was no statistically significant difference between groups considering the type of treatment.

On average, the participants in the active and Sham groups initially had depressive symptoms considered moderate (BDIi = 28.54 ± 5.51 vs. 27.31 ± 7.29). At the end of the third assessment, the initial BDI score decreased 35,0% in the active group and 45,99% in the sham group. In the fourth assessment, this initial BDI score decreased by 43.93% vs. 44.67% in the active vs. Sham groups, respectively (BDIiv = 16 ± 8.13 vs. 15.11 ± 11.42; ΔBDIiv – i = −12.54 vs. −12.20), with the participants being reclassified, on average, as having minimal or mild depressive symptoms. Generalized Estimating Equations (GEE) factor analysis showed that there was an influence of the time factor on this improvement (*p* < 0.001), but not of the interaction time vs. type of stimulation (*p* = 0.93) or the type of stimulation factor alone (*p* = 0.42). Regarding the evaluation performed 1 year after the end of treatment, the tDCSs group showed an increase of 4.19 points on the BDI-II scale (from 15.11 to 19.30 points), while the tDCSa group increased on average only 1 point (from 16 to 17 points) (Figure 2 and Table 8). There was no statistically significant difference between groups. When analyzing the effect of the type of treatment over time on BDI variation, controlling for the covariables age, sex, education, AED, DAD, occupational situation, and income, we observed that there was no significant change in the result found, as confirmed by the correlation test that showed no correlation between these covariables and the deltaBDI (final-initial).

**TABLE 8:** Effect of treatment on depressive symptoms, quality of life, and anxiety.

	Mean (DP)					Delta % (D-A)	*p* value
	Day 1 (A)	Day 15 (B)	Day 30 (C)	Day 60 (D)	Follow up (E)		
tDCSa	*n* = 13	*n* = 11	*n* = 12	*n* = 9	*n* = 10		
tDCSs	*n* = 13	*n* = 9	*n* = 11	*n* = 10	*n* = 8		
**BDI-II**							
tDCSa	28.5 ± 5.5	23.0 ± 10.6	18.5 ± 9.9	16.0 ± 8.1	17.0 ± 8.8	−43.93%	
tDCSs	27.3 ± 7.2	19.8 ± 7.4	13.9 ± 9.3	15.1 ± 11.4	19.3 ± 8.2	−44.67%	
Intervention effect							0.42
Time							< 0.001
Interaction time*Intervention							0.93
QOLIE-31							
tDCSa	47.7 ± 13.0	–	58.1 ± 17.1	59.8 ± 17.7	62.8 ± 20.9	25.51%	
tDCSs	50.5 ± 13.5	–	58.4 ± 16.5	61.1 ± 11.8	59.6 ± 13.0	20.91%	
Intervention effect							0.9
Time							0.003
Interaction time*Intervention							0.92
HAM-A							
tDCSa	23.3 ± 8.3	–	21.2 ± 8.3	20.8 ± 10.2	–	−10.07%	
tDCSs	22.9 ± 4.2	–	23.8 ± 7.5	17.7 ± 6.1	–	−22.42%	
Intervention effect							0.86
Time							0.05
Interaction time*Intervention							0.09

*Data were analyzed through Generalized Estimating Equations with a dependent variable (BDI-II, QOLIE-31, or HAM-A score), within-subject variable (time) and variable between subjects (active × Sham tDCS). Bonferroni was the post hoc analysis.*

### Quality of Life

**FIGURE 3:**
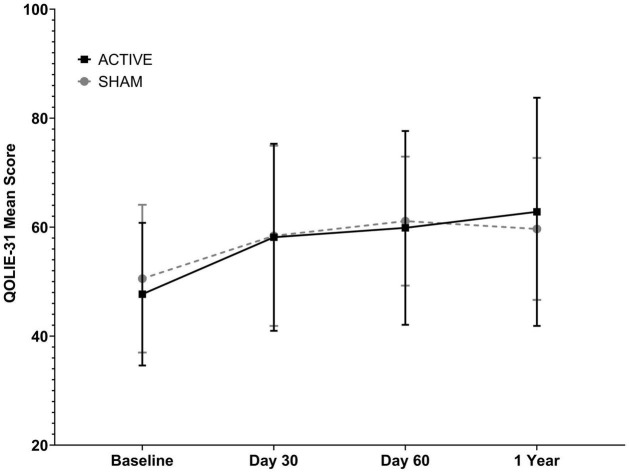
Quality of life assessment in the Sham and Active groups using the Epilepsy Quality of Life Inventory (QOLIE-31) in three stages: (i) pre-treatment; (ii) after 20 daily sessions; (iii) after 1 month performing tDCS once a week for 3 weeks. Data are reported as mean +SD QOLIE-31 score. Data analysis was performed using the Generalized Estimation Equations (GEE) model. There was no statistically significant difference between groups considering the type of treatment.

Likewise, the quality of life of the participants improved over time in both groups (*p* = 0.003, GEE analysis), with no statistically significant influence of the type of treatment on this improvement (*p* = 0.9), of the interaction between the time and the factor or the type of stimulation received (*p* = 0.92). The increase in the score of the QOLIE-31 questionnaire in the active and Sham groups was ΔQOLIE31iii-i = 25.51% vs. 20.91%, respectively. Data about the mean QOLIE31 mean points in each evaluation are available in Table 8. The evaluation carried out about 1 year after the end of the treatment did not show statistically significant changes in Quality of Life in relation to the third evaluation in either group (Figure 3 and Table 8).

### Anxiety

**FIGURE 4:**
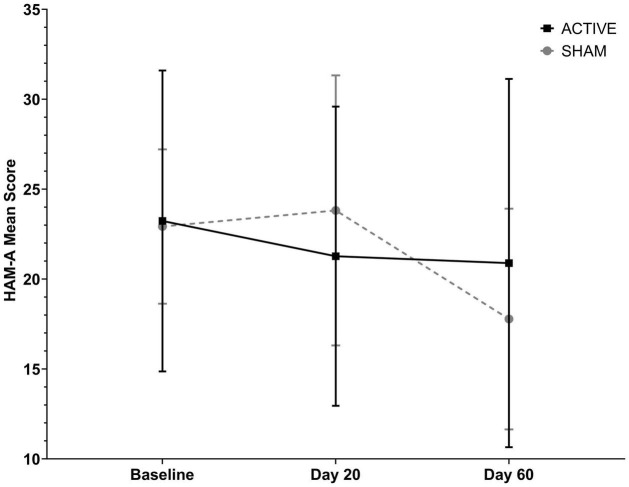
Anxiety symptoms assessed in the active tDCS and Sham groups using the Hamilton Anxiety Scale (HAM-A) in three stages: (i) pre-treatment; (ii) after 20 daily sessions; (iii) after 1 month of follow-up, performing tDCS once a week for 3 weeks. Data are reported as mean +SD. Data analysis was performed using the Generalized Estimation Equations (GEE) model. There was no statistically significant difference between groups considering the type of treatment.

Anxious symptoms were evaluated in three stages on days 1, 30 and 60, using the score obtained with the HAM-A questionnaire. In relation to the initial assessment, the last anxiety assessment showed a reduction of 2.34 points (from 23.23 ± 8.36 to 20.89 ± 10.24) or 10.07% in the tDCSa group, and a reduction of 5.14 points in the tDCSs group (from 22.92 ± 4.28 to 17.78 ± 6.14), representing a reduction of 22.42% (Figure 4 and Table 8). The GEE analysis revealed an influence of the time factor on this improvement (*p* = 0.05), but not of the time*type of stimulation interaction (*p* = 0.09) or the type of stimulation factor alone (*p* = 0.86). There was no statistically significant difference between groups. Analysis of the effect of the type of treatment over time on the BDI variation, with control for the covariables age, sex, education, AED, DAD, occupational situation and income, revealed no statistically significant change in the results.

## Discussion

In this study, we proposed the application of 20 daily sessions of tDCS at home followed by 3 weekly sessions at the research center in order to reduce depressive and anxious symptoms and to improve the quality of life of patients with PWE. The anode was positioned over the left DLPFC and the cathode over the right DLPFC (bimodal stimulation).

Participants in the active tDCS group reported more moderate and severe local adverse effects than participants who received Sham stimulation, although the difference between groups was non-significant. Only 3 participants dropped out of the study (1 from the Sham group and 2 from the active group). On average, both groups performed 13 effective stimulations at home (out of a total of 20) and 2 of 3 sessions in the laboratory, reflecting limitations in adherence to the proposed protocol.

Some of the reasons why patients with epilepsy do not adhere to the self-administered tDCS treatment may be accidental, due to forgetfulness or uncertainty about the doctor’s recommendations, or may be intentional, due to local adverse effects or due to their own treatment expectations (Eatock and Baker, 2007; Das et al., 2018). Remote patient supervision via social media (Whatsapp) during treatment might help on these venues, but it was not done as a routine in our study. However, it was available as requested by the participants. The research team did not send daily messages or frequent messages to the participants reminding them about the use of the equipment, a fact that may have compromised treatment adherence. Routine daily messages about the treatment perhaps should be considered in future trials, as it may improve the adherence of the patients to the study protocol. Treatment adherence is a health problem for patients with epilepsy (DiIorio et al., 2004; Ferrari et al., 2013). For efficacy studies like ours we emphasize the importance of maintaining a more frequent, standardized and close monitoring of the study participants to ensure compliance with the proposed protocol.

There were two possible points of concern about the safety of applying this bimodal protocol to the sample studied: (i) 76.9% of the participants in active group had at least one crisis in the last year (uncontrolled epilepsy); (ii) many patients had an epileptogenic focus on the left brain hemisphere, where the anodal (excitatory) stimulation was performed. Nevertheless, the program of approximately 15 sessions was safe for the sample studied. There was no statistically significant difference in the frequency of seizures before and after the beginning of treatment in either group. One participant had 1 epilepsy crisis during the 30 days prior to the study, 1 epilepsy crisis during the 30 days of home treatment and 6 epilepsy crisis during the 30 and 60 days of the study, but it is not possible to establish a direct relationship with the stimulation performed considering that the other participants showed no increase in the frequency of epilepsy crisis.

Regarding quality of life and depressive and anxious symptoms, even controlling for confounding or interaction variables, both groups showed a similar improvement in symptoms over time, but not due to the influence of the type of treatment. From a pharmacological point of view, antiepileptic drugs, including sodium or calcium channel blockers, and medications that influence neurotransmitters such as GABA, may impact the aftereffects of tDCS (Nitsche et al., 2003; McLaren et al., 2018). Another issue that may have influenced the effect of tDCS was that, considering that about 90% of the participants had an epileptogenic focus on the left, the bimodal stimulation with anode on the left and cathode on the right side may have not been the best configuration of transcranial stimulation for the studied sample. In order to reduce the imbalance of interhemispheric activity, maybe it could be interesting to study cathodal stimulation on the left and anodal on the right DLPFC for the participants that joined our study, considering that most of them had left temporal lobe epilepsy.

One possible explanation for the similarity of response between the two groups is the high significance of the placebo effect in studies on treatment of depression and anxiety (Rutherford and Roose, 2013; Kirsch, 2019). The placebo effect may have overshadowed the active effect of tDCS. The placebo effect can be generated by conditioning mechanisms, especially when dealing with ingested drugs, or by expectations in the case of non-pharmacological interventions, such as tDCS (Stewart-Williams and Podd, 2004). At the time of recruitment, all participants heard statements such as “this device serves to treat depressive and anxiety symptoms and to improve the quality of life”, which can be suggestive and can be involved in the placebo effect.

In the thesis “Placebo’s Feats and Effects,” Saretta highlights the differentiation between “illness” and “disease” as a fundamental criterion for the understanding of the placebo phenomenon in clinical trials (Saretta, 2018). According to the author, the improvement caused by the placebo treatment would be on “illness,” considered as the human experience of the disease, while the active principle of treatment would act on “disease,” the biological mechanism of the pathology. Devices like tDCS probably offer both biological and placebo effects. Patients with epilepsy have a very impaired psychosocial context which results in reduced quality of life and worsens the experience of these patients with the disease (“illness”). Thus, health interventions have a significant impact on “illness” in this population, partially explaining the observed placebo effect. The frequent interaction between the participant and the researcher may have acted as a small social intervention on harmful environmental factors such as isolation and social and relationship problems (Müller and Gomes, 2007). There are many types of social intervention that are effective for the improvement of psychiatric disorders (Nagy and Moore, 2017).

In a review article about the needs, perspectives and perceptions of PWE regarding the Health Care System, Muller & Gomes have shown that there are important financial, social, and relationship problems in this population. With regard to social adjustment, difficulty in interpersonal relationships was relatively common; 42% of patients reported having only few or no friends, 27% of female patients considered their crises to be responsible for the difficulties of family life, 40% of the patients reported that they did not have an affective relationship, and 45% reported having difficulty in establishing this type of relationship. Due to the disease itself, 10% of patients reported being involved in legal disputes (Müller and Gomes, 2007). The different social relationship variables, such as social integration, social support and negative interaction, are associated with health outcomes (Cohen, 2004; Uchino, 2006; Aragão et al., 2018).

In our study, the researcher-participant interaction through frequent monitoring via social media *(Whatsapp)* during the intervention period, interviews about psychosocial content and application of a physical intervention (tDCS) may act as a social intervention and can explain in part the clinical improvement observed in both groups. Around the world, many types of social and educational interventions are being developed as effective interventions for the improvement of psychiatric disorders and quality of life (Elafros et al., 2013; Nagy and Moore, 2017; He et al., 2019; Mathias et al., 2020; Pandey et al., 2020). These models of care provided by a multidisciplinary team specialized in epilepsy can improve the quality of care provided when compared to exclusive medical care, since they provide greater availability of time for care if they are divided among professionals.

Previous works using tDCS to treat depression in epilepsy were conducted, with different results (Liu et al., 2016; Azmoodeh et al., 2021). Liu et al. (2016) performed a double-blinded, sham-controlled, randomized, parallel-group study of 5 days of fixed-dose (2 mA, 20 min) tDCS for treating depression and memory dysfunction in patients with temporal lobe epilepsy (TLE). These authors observed only a temporary improvement in the depression scores when compared with the sham group. Azmoodeh et al. (2021) did a prospective controlled study in which the intervention was performed in 10 sessions of 20 min. They observed that tDCS treatment decrease depression, anxiety, and stress in patients with epilepsy. We did not observe these effects. However, our results are in line with these studies regarding safety of the tDCS in patients with epilepsy. Two main methodologic differences between our study and these two studies are: (a) we propose to study the effect of tDCS on depressive symptoms considered at least mild (minimum BDI score of 14 points), and (b) we propose to expand the treatment of depressive symptoms of patients with TLE using 23 sessions of bimodal tDCS (anode positioned over the left DLPFC and cathode over the right DLPFC). In order to make the prolonged use of the device by the participants viable, we propose the innovative use of the home-based and self-administered tDCS. In our view, these methodological differences may explain, at least in part, the differences and the placebo effect that we observed in our study and that was not observed in these two previous studies.

Our study as limitations and we recognize it. One of them is the limited adherence of patients to the treatment. Despite this may have compromised the effectiveness of the study, it provided us with valuable information about the external applicability and effectiveness of tDCS in treatment of psychiatric comorbidities in epilepsy. Also, this study was not remotely supervised, only monitored and it may have influenced anxiety and depression in our patients. However, it has some strength that need also be recognized. It was double-blind, randomized, sham-controlled clinical trial done a team that was equally trained. Also, psychiatric diagnose were based in structured questionnaires, which contribute to increase the validity of the study.

Concluding, we observed limitations in adherence to the proposed treatment, both regarding the routine use of the equipment and the visits to the research service for evaluation. It is necessary to structure a frequent monitoring of participants. There were expressive improvements in quality of life, depressive and anxious symptoms in both groups. One possible explanation for the similarity of response between the two groups is the high significance of the placebo effect in studies on treatment of depression and anxiety, overshadowing the tDCS effects. In addition, antiepileptic drugs may impair tDCS effects and, considering that 90% of the participants had an epileptogenic focus on the left, perhaps bimodal stimulation with anode on the left side and cathode on the right was not the best configuration of transcranial stimulation for treatment of depressive symptoms in the studied sample.

## Data Availability Statement

The raw data supporting the conclusions of this article will be made available by the authors, without undue reservation.

## Ethics Statement

The studies involving human participants were reviewed and approved by Committee of the Hospital de Clínicas de Porto Alegre (#CAAE 83801517100005327). The patients/participants provided their written informed consent to participate in this study.

## Author Contributions

SM, WC, and MB: conception and design of the work. LA, PR, CT, JB, and PS: acquisition of data. SM, LA, WC, RB, TS, and MB: analysis and interpretation of data. SM, LA, WC, and MB: drafting the work. SM, LA, PR, CT, JB, RB, TS, PS, WC, and MB: revising the manuscript final approval of the version to be published. All authors contributed to the article and approved the submitted version.

## Conflict of Interest

The authors declare that the research was conducted in the absence of any commercial or financial relationships that could be construed as a potential conflict of interest.

## Publisher’s Note

All claims expressed in this article are solely those of the authors and do not necessarily represent those of their affiliated organizations, or those of the publisher, the editors and the reviewers. Any product that may be evaluated in this article, or claim that may be made by its manufacturer, is not guaranteed or endorsed by the publisher.

## Footnotes

1
https://www.jove.com/video/57614/home-based development of current stimulation devices transcranial direct
